# High-resolution intravascular magnetic resonance quantification of atherosclerotic plaque at 3T

**DOI:** 10.1186/1532-429X-14-20

**Published:** 2012-03-26

**Authors:** Di Qian, Paul A Bottomley

**Affiliations:** 1 Division of MR Research, Department of Radiology and Radiological Science, Johns Hopkins University, Baltimore, MD, USA; 2 Division of MR Research, Department of Radiology and Radiological Science, Johns Hopkins School of Medicine, 600 N Wolfe St, Park 310, Baltimore, MD, USA

**Keywords:** Interventional MR, Intravascular MR, Atherosclerosis, Fibrous cap thickness, High-resolution

## Abstract

**Background:**

The thickness of fibrous caps (FCT) of atherosclerotic lesions is a critical factor affecting plaque vulnerability to rupture. This study tests whether 3 Tesla high-resolution intravascular cardiovascular magnetic resonance (CMR) employing tiny loopless detectors can identify lesions and accurately measure FCT in human arterial specimens, and whether such an approach is feasible *in vivo *using animal models.

**Methods:**

Receive-only 2.2 mm and 0.8 mm diameter intravascular loopless CMR detectors were fabricated for a clinical 3 Tesla MR scanner, and the absolute signal-to-noise ratio determined. The detectors were applied in a two-step protocol comprised of CMR angiography to identify atherosclerotic lesions, followed by high-resolution CMR to characterize FCT, lesion size, and/or vessel wall thickness. The protocol was applied in fresh human iliac and carotid artery specimens in a human-equivalent saline bath. Mean FCT measured by 80 μm intravascular CMR was compared with histology of the same sections. *In vivo *studies compared aortic wall thickness and plaque size in healthy and hyperlipidemic rabbit models, with post-mortem histology.

**Results:**

Histology confirmed plaques in human specimens, with calcifications appearing as signal voids. Mean FCT agreed with histological measurements within 13% on average (correlation coefficient, *R *= 0.98; Bland-Altman analysis, -1.3 ± 68.9 μm). *In vivo *aortic wall and plaque size measured by 80 μm intravascular CMR agreed with histology.

**Conclusion:**

Intravascular 3T CMR with loopless detectors can both locate atherosclerotic lesions, and accurately measure FCT at high-resolution in a strategy that appears feasible *in vivo*. The approach shows promise for quantifying vulnerable plaque for evaluating experimental therapies.

## Background

Atherosclerosis is characterized in its advanced stages by lesions containing extra-cellular lipids, foam cells and/or calcium deposits in the arterial wall, covered by fibrous caps [1-3]. Its clinical symptoms often involve progressive myocardial, cerebral or peripheral ischemia resulting from the ensuing stenosis, but lesion rupture can cause acute thrombosis, stroke, heart attack and death [1-5]. Fibrous cap thickness (FCT) is an important factor in determining the vulnerability of atherosclerotic plaques. Because thin caps are associated with large plaque stress and the likelihood of rupture [3-6], an ability to accurately quantify FCT *in situ *could prove key to identifying plaques that are most vulnerable to rupture and would benefit most from intervention, as well as evaluating the mechanistic and morphological changes responsive to therapies aimed at plaque regression.

While an FCT of less than 65 μm has been used to classify fibrous caps in coronary plaques as vulnerable [7-10], currently there isn't an accepted clinical methodology for identifying unstable plaques in patients *in situ *[11]. X-ray angiography routinely detects stenoses by imaging the arterial lumen, but not the vessel wall or lesion morphology *per se*, nor early-stage lesions with mild-to-moderate stenoses [5]. Intravascular ultrasound (IVUS) can provide trans-luminal imaging at ≥100 μm resolution without ionizing radiation [9,11,12], although it is typically performed during withdrawal of an imaging catheter threaded over a guidewire inserted under X-ray fluoroscopy [13]. IVUS studies of human endarterectomy specimens suggest that an FCT threshold of 650 μm can discriminate symptomatic from asymptomatic carotid artery stenoses [14]. Optical coherence tomography (OCT) is another intravascular imaging option [15] that is fast, has high resolution (10-20 μm), but a limited penetration depth of about 1 mm [9,10,15,16]. It also requires a blood-free environment, afforded by intermittent saline flushes through an X-ray fluoroscopic guide catheter [9,10].

Cardiovascular magnetic resonance (CMR) offers the multi-functionality and intrinsically high soft-tissue contrast that could potentially add unique value and provide intravascular imaging without X-ray guidance or ionizing radiation [17-20]. However, when applied noninvasively with external detector coils, CMR's spatial resolution for differentiating FCT is more limited (> 200 μm) than both IVUS and OCT [17-20]. To improve local sensitivity and spatial resolution, intravascular CMR employing tiny detectors as guidewires or catheters was proposed many years ago [21-26]. At clinical field strengths of B_0 _= 1.5 Tesla (T), these devices have demonstrated efficacy in characterizing vessel wall thickness and plaque components in larger vessels with in-plane resolution ≥100 μm [27-30]. Nevertheless, as emphasized in recent editorials, the speed and spatial resolution of intravascular CMR remain a challenge [31,32]. A resolution of 80 μm or better may be needed to resolve plaque components in smaller critical vessels, and at this time it is not known whether an intravascular CMR modality will ever be a practical tool for plaque characterization or FCT measurements in patients [31].

Yet the recent emergence of clinical 3T MR scanners does afford some new opportunity for addressing these problems by virtue of an almost quadratic gain in signal-to-noise ratio (SNR) with B_0_, due to the intrinsic noise properties of tiny detectors [33,34]. As a consequence, "loopless antenna" detectors [25] realizing a ~4-fold higher SNR and a ~10-fold increase in visual field-of-view (FOV) at 3 T compared to the original 1.5 T detectors, have been demonstrated [33]. This, with local heating controlled at ≤1°C [33]. In the present study, we investigate whether such 1-2 mm diameter detect-only 3 T intravascular antenna guidewires can both identify atherosclerotic lesions without X-ray fluoroscopic guidance, and provide high-resolution (< 100 μm) imaging of vessel anatomy and accurate FCT and wall thickness measurements in a conventional clinical 3T MR scanner. FCT is measured at 80 μm resolution in diseased human arterial specimens *in vitro*, and the results compared with histology. The feasibility of the approach is then demonstrated *in vivo* in healthy and hyperlipidemic rabbit aortas, a vessel that is comparable in size to a human coronary.

## Methods

### Devices

**Figure 1:**
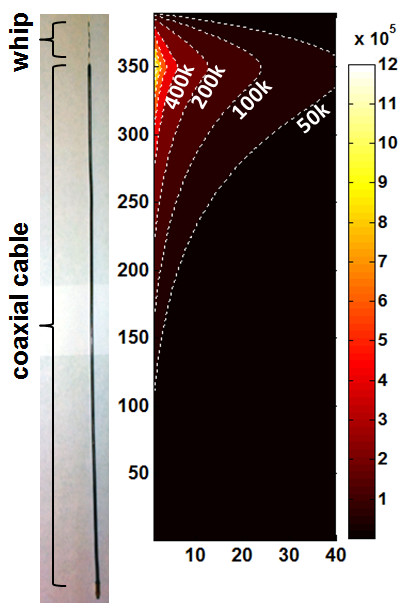
**Photograph of semi-rigid, 40-cm cable-length, loopless CMR detector used for *in vitro *studies (left), and its absolute SNR distribution (in ml^-1^Hz^1/2^; contours and color-coded at right) on a one-sided plane along its length**. Distances are in mm with the probe oriented parallel to the main magnetic field (z-axis). The SNR is cylindrically symmetric about the long axis.

*In vitro *CMR studies were performed with a 3T loopless "receive-only" detector fabricated from a UT-85C (outer diameter, OD = 2.2 mm) semi-rigid copper coaxial cable (Micro-coax, Inc., Pottstown, PA) to fit within the approximately 8 - 15 mm lumens of human vessel specimens studied (Figure 1). The length of the detector's coaxial cable was tuned to a quarter-wavelength (λ/4 ≈ 40 cm). The central conductor was extended an additional 4 cm beyond the cable portion to form a whip that maximized signal at the whip-cable junction [33]. The outer surface of the cable was insulated with 0.02 mm polyester (dielectric constant, ε = 3) heat-shrink tubing (Advanced Polymer Inc, Salem, NH). To determine its sensitivity in the plane of the cable for angiography and/or tracking, the absolute SNR of the device in a 0.35% saline phantom with radio-frequency (RF) electrical properties comparable to those of tissue (conductivity, σ = 0.6 S/m, dielectric constant ε = 80) was determined by the numerical method-of-moments (FEKO EM analysis software, Stellenbosch, South Africa) from the ratio of the circularly polarized RF field excited by a 1A current, to the square root of the antenna resistance in the saline [33]. Since the SNR is cylindrically symmetric about the cable's long axis, the computation was performed on a single plane on one side of the detector, over the range 0.1 ≤ *r ≤*40 mm from the detector outer conductor, and 0 ≤ *z ≤*39 cm from the detector tip, as well as in axial planes about the detector junction.

**Figure 2:**
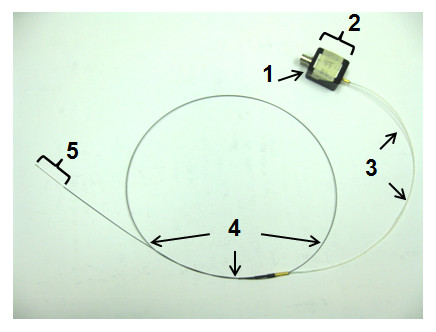
**Photograph of the biocompatible 3T loopless CMR detector**. The components are: (1) BNC connection to the MR scanner interface; (2) tuning, matching, and decoupling circuit box; (3) coaxial extension cable; (4) coaxial cable from an obsolete 1.5T Intercept guidewire (OD = 0.76 mm); and (5) the extended inner conductor whip (length = 4 cm).

*In *vivo experiments were performed with a biocompatible nitinol loopless imaging detector (Figure 2) with the same coaxial structure as the detector used for the *in vitro *experiments. It was formed by modifying an obsolete 0.76 mm OD *Intercept *1.5T guidewire (MRI Interventions, formerly Surgi-Vision Inc, Memphis TN) for 3T use by retuning the whip to 40 mm, and extending the cable length to 3λ/4 (for a total cable length of 120 cm) to permit practical *in vivo *use in the magnet bore. Device heating during CMR was controlled at ≤1°C with decoupling circuitry described previously, and demonstrated in safety studies on a saline gel phantom [33]. The detector was tuned, matched, and interfaced to a 3T Philips Achieva MR scanner equipped with an X-ray C-arm (Philips Healthcare, Cleveland, OH) [33].

### *In vitro *Studies

The 3T loopless CMR detector was first used to identify suspected plaques inside a fresh ~10 cm-long human iliac artery specimen, complete with bifurcation, harvested at autopsy within 3 hours of death. All studies of decedent specimens were approved by this institution's Office of Human Subject Research, Institutional Review Board. The vessel was secured to a plastic frame inside a 0.35% saline phantom (with σ = 0.6 S/m, ε = 80). The 3T detector was inserted into the lumen, and a spin-lattice relaxation time (T_1_)-weighted angiographic scan performed in the coronal plane along the luminal long-axis (three-dimensional fast field echo, 3D FFE; FOV = 120 x73 mm; resolution, 0.5 x0.5 x1 mm; array size = 256 × 256 × 10; repetition time/echo time, TR/TE = 25/2.6 ms; flip-angle, FA = 20°; scan time = 143 s). After locating suspected plaques on the coronal angiographic scans, high-resolution trans-axial spin-spin relaxation time (T_2_)-weighted scanning of the vessel wall was performed (3D turbo spin-echo, TSE factor = 9; FOV = 20 × 20 mm; TR/TE = 1500/32 ms; resolution = 80 μm × 80 μm × 2 mm; FA = 90°; 4 averages; scan time = 1514 s) to reveal the fibrous cap. To determine the effect of image resolution on measurements of FCT, the same section of a human iliac specimen was imaged with the same sequence but at 80, 100 and 140 μm in-plane resolution.

The locations of the high-resolution trans-axial images were registered to the vessels by first measuring their distance to the ends of the vessels as visualized on the coronal images. After CMR, vessels were decalcified for 48 hrs and cut into 5-10 mm sections, each facing the location of the corresponding trans-axial image to the nearest millimeter. The histological sections were visually inspected for antenna-related injury to the vessel wall, embedded in wax, microtomed, and stained with hematoxylin and eosin (H&E) at this institution's pathology service. The sections were then photographed with a microscope-mounted digital camera.

To test whether high-resolution 3T interventional CMR with a loopless detector could be used to provide accurate measures of FCT, thirty-one diseased carotid artery segments with atherosclerotic lesions identified by palpation, were harvested from sixteen adult human cadavers. These vessel segments were fixed in 10% formalin at 3°C for 24 hr. High resolution T_2_-weighted CMR of the vessel wall was performed on vessels mounted in the saline phantom, followed by sectioning and histology as described above.

The FCT was measured in both the CMR and the histology sections between the two shoulder ends of the fibrous cap at an interval of 480 μm, or at about every 6 pixels in the 80 μm CMR. Distance was measured in histology sections using a movable scale overlaid on the digital micrograph, and in CMR by counting pixels and multiplying by the resolution. Measurements were restricted only to the regions with clear fibrous cap and lumen borders. The average of all of the FCT measurements from each image was recorded as the mean FCT for that image section. The mean FCT for the iliac specimen imaged at 80, 100 and 140 μm resolution was measured the same way.

### *In vivo *Studies

High-resolution intravascular CMR was performed *in vivo *using the 3T biocompatible loopless detector in a healthy adult New Zealand white rabbit, and a 30-month old male Watanabe heritable hyper-lipidemic (WHHL) rabbit which is prone to spontaneously develop atherosclerotic lesions. The study was approved by this institution's Animal Care and Use Committee. The animals were sedated and positioned supine in the 3T scanner, and the detector inserted into the aorta via the femoral artery and a 5-French introducer under CMR guidance. The same T_1_-weighted angiographic coronal scan as in the *in vitro *studies was used, except that the scans were cardiac-triggered. The location of the detector's junction in the animal relative to the renal bifurcation was determined by X-ray fluoroscopy and on the coronal T_1 _angiographic images. The locations of the high-resolution trans-axial images relative to the detector's junction and to the center of the renal bifurcation were noted for subsequent co-registration.

High-resolution trans-axial CMR of the aortic wall was performed above the renal bifurcation. A T_2_-weighted TSE sequence modified for black-blood with cardiac gating was used for the healthy rabbit (FOV = 80 × 40 mm; TR/TE = 3000/21 ms; resolution = 80 μm × 80 μm × 3 mm; array size = 1008 × 1008; FA: 90°; 4 averages; scan time = 597 s), and a 3D T_1 _weighted TSE black-blood sequence with shortened scan-time was used for the hyperlipidemic rabbit (FOV = 20 x10 mm, TR/TE = 429/26; resolution = 80 μm × 80 μm × 2 mm; array size = 256 × 256 × 10; FA: 90°; 8 averages; scan time = 436 s). After CMR, the rabbits were humanely sacrificed. The aortas were harvested and cut into 5 mm sections facing the locations at which trans-axial CMR was performed, referenced relative to the renal bifurcation. The sections were visually inspected for injury, then H&E stained for histology as above.

## Results

### *In vitro *Studies

The computed absolute SNR distribution of the loopless detector is shown in Figure 1. The highest SNR occurs at the detector's whip-cable junction at a radial distance *r = *0.1 mm, falling to 17% of the peak at *r = *5 mm. However, excluding the tip [35], the absolute SNR of the device for small *r *is preserved to at least half of the peak value over an extended region in the longitudinal plane for distances *z ≤*80 mm from the device junction.

**Figure 3:**
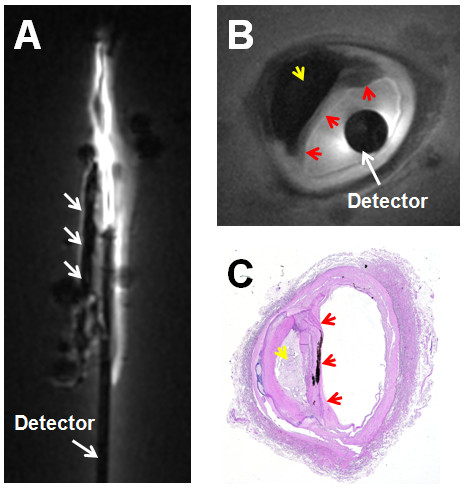
**(A) T_1_-weighted angiographic coronal image of a human iliac artery specimen *in vitro *showing calcified plaque inside vessel wall (3 parallel arrows, center)**. The detector's cable appears dark, with a bright signal surrounding the narrower whip. (B) High resolution intravascular 3 T CMR of the vessel wall cross section showing calcified plaque (yellow arrow), fibrous cap (red arrows) and the circular cross-section of the detector (white arrow). (C) Histological image of the iliac artery vessel wall showing calcified plaque (yellow arrow) and fibrous cap (red arrows).

Figure 3 exemplifies T_1_-weighted angiographic scout CMR, and high-resolution trans-axial images acquired from human iliac artery specimens *in vitro*. In coronal angiographic images, calcified plaques appeared as regions of low intensity or signal voids, such as those between the vessel walls below the iliac bifurcation in Figure 3(A). The guide-wire CMR detector itself contains no mobile protons and also appears as a signal void: the absence of any image decoupling artifact around the probe attests to the efficacy of the tune/match/decoupling circuitry [33,35]. The extended region of sensitivity permits high-resolution imaging virtually anywhere in these lesions, and the trans-axial 80 μm image of the iliac vessel wall (Figure 3B) reveals a calcified plaque as a void separated from the fluid-filled lumen by the fibrous cap. The vessel wall morphology is confirmed by microscopy of the corresponding histological section (Figure 3C).

**Figure 4:**
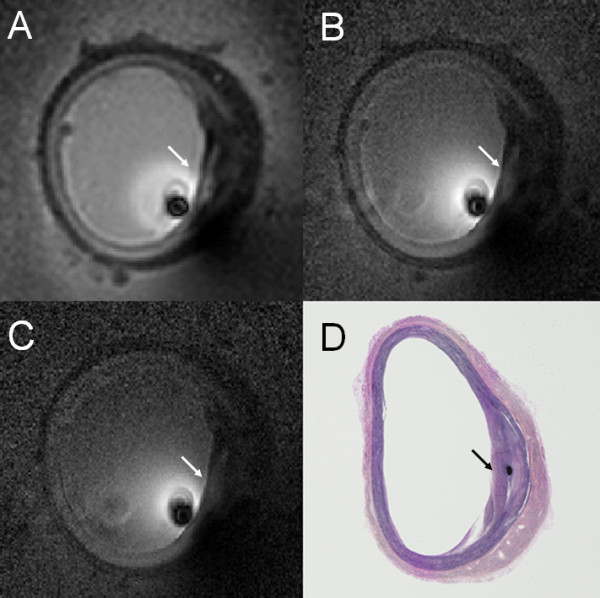
**High resolution CMR of human iliac artery specimen acquired using the 3 T loopless CMR detector showing a fibrous cap (arrows) at (A) 140 μm; (B) 100 μm; and (C) 80 μm resolution (here, image pixel intensity is inversely scaled to compensate for the ~*1/r *signal drop with distance, *r*, from the detector)**. The corresponding histological section is shown in (D).

CMR of a human iliac vessel acquired at 80, 100, and 140 μm resolution is presented in Figure 4 with corresponding histology. Mean FCT was 360, 243, and 206 μm, when measured with 140, 100, and 80 μm resolution intravascular CMR, respectively (Figure 4A-C). Mean FCT from histology was lower at 186 μm (Figure 4D), consistent with the view that reducing the CMR spatial resolution (increasing voxel size) decreases the accuracy of FCT measurements.

**Figure 5:**
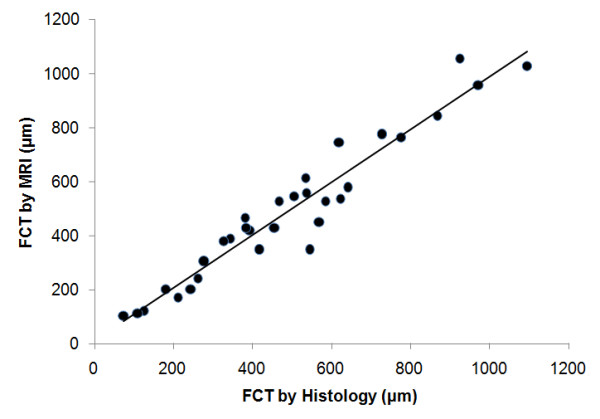
**Comparison of mean FCT measured by high-resolution intravascular 3 T CMR and by histology**. The line of best fit for all the data (n = 31) is y = 0.98 × + 11.5 μm (correlation coefficient, R = 0.96; probability that difference in FCT is insignificant, P = 0.99).

**Figure 6:**
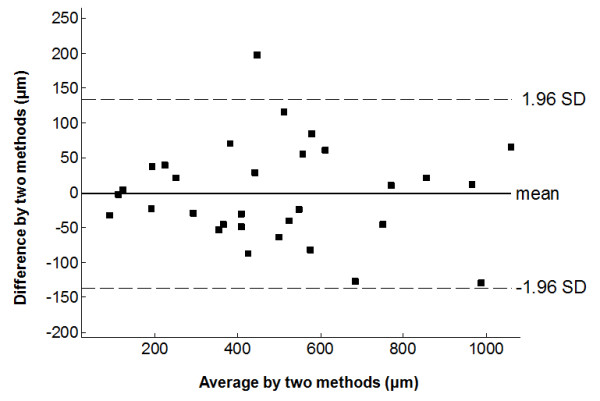
**Bland-Altman plot for the mean FCT measured by high-resolution CMR and by histology**.

All 31 vessel segments exhibited lesions with fibrous caps based on histology. High-resolution CMR of carotid vessel walls at 80 μm also revealed atheroma in all vessel segments, permitting 3-13 individual FCT measurements from each CMR section, and 3-12 FCT measurements in each of the corresponding histological sections, from which the mean FCT was determined. The mean FCT measured from CMR is plotted against the mean FCT from histology in Figure 5. The mean FCT by histology ranged from 74 μm to 1.1 mm. The two sets of measurements are highly correlated (correlation coefficient, R = 0.96). Even limiting the data to those samples with mean FCT < 650 μm (24 out of 31 samples) as measured by histology, preserves the correlation (y = 0.921x + 29.8, R = 0.91, P = 0.98). Bland-Altman analysis (Figure 6) shows excellent agreement between the FCT measured by CMR and that measured by histology (-1.3 ± 68.9 μm).

### *In vivo *Studies

**Figure 7:**
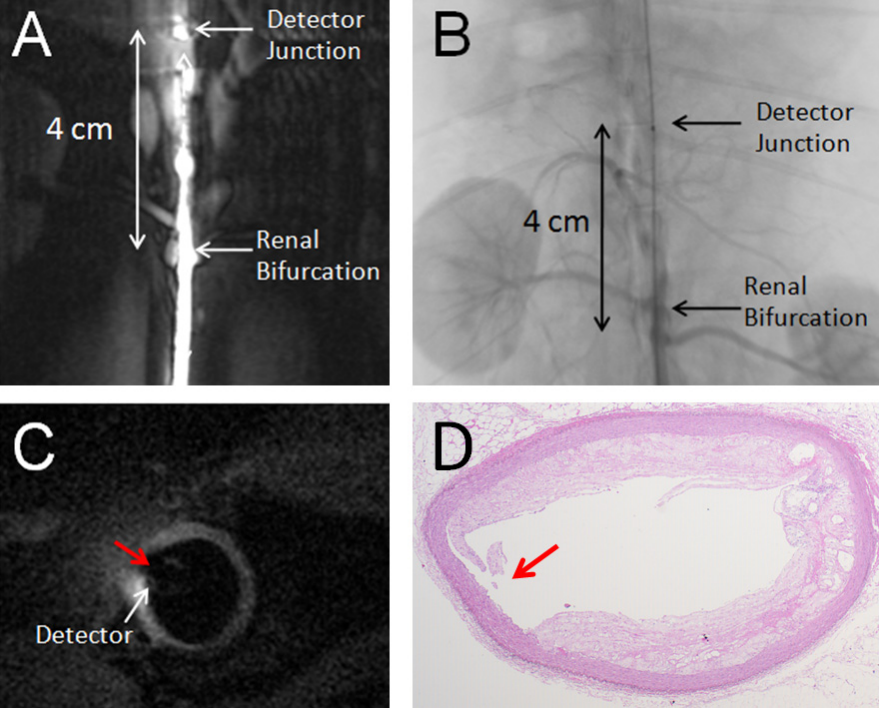
**(A) *In vivo *T_1_-weighted angiographic coronal image acquired by loopless intravascular CMR detector from a female New Zealand white rabbit showing the aorta near the renal bifurcation**. (B) X-Ray fluoroscopic image confirming placement of the loopless CMR detector in the aorta 4 cm above the renal bifurcation. (C) The cardiac-gated 80 μm high-resolution 3 T CMR of the rabbit aortic vessel wall 1 cm above the renal bifurcation. (D) Post-mortem histological image of aortic vessel wall of the same imaging slice. The shape is distorted during harvesting and staining processes. A small indent is evident on the vessel wall in both the image (C) and histology (D; red arrows)

The placement of a biocompatible 3T loopless detector in the New Zealand White rabbit aorta in vivo under CMR angiography is exemplified in Figure 7(A). This shows the detector's whip-cable junction where signal intensity is highest (Figure 1), 4 cm above the renal bifurcation. Unlike CMR endoscopic probes [33,34], the receive-only intravascular detector used here reveals the entire aorta and some adjacent anatomical structures, including the renal bifurcation. X-ray fluoroscopy confirmed the location of the whip-cable junction 4 cm above the renal-bifurcation (Figure 7B). An 80 μm trans-axial high-resolution CMR of the aorta delineates the vessel wall in the rabbit (Figure 7C). The vessel wall thickness was measured from the image at 12 equi-angular locations around the vessel, commencing at a vessel feature identifiable on both CMR and histological images (solid arrows, Figure 7C, D). The average wall thickness from high-resolution intravascular 3T CMR was 0.54 mm ± 0.06 (SD) (Figure 7C), in agreement with an average thickness of 0.55 ± 0.07 mm measured the same way in the histological section (Figure 7D).

**Figure 8:**
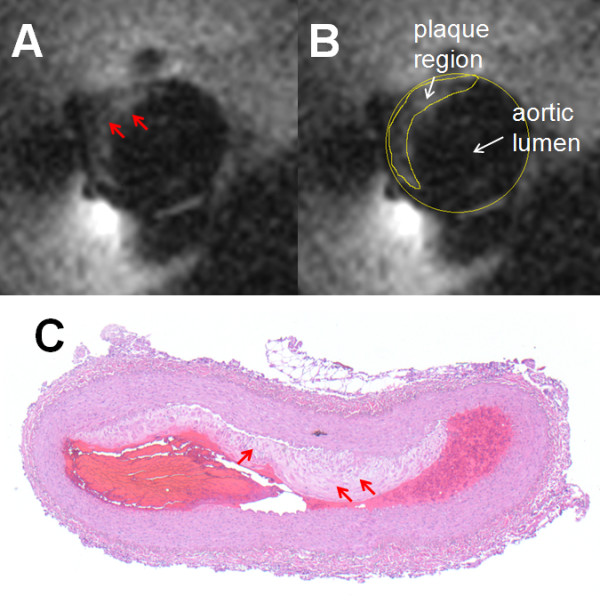
**(A) *In vivo *cardiac-gated T_1_-weighted 80 μm resolution axial 3 T image acquired from a male hyperlipidemic rabbit aorta showing an area of abnormal tissue extending from the inner tissue wall (red arrows), 1 cm above the renal bifurcation**. (B) The plaque region outlined from the vessel cross-section for quantification. (C) Histology of the corresponding vessel cross-section (collapsed during processing) showing an atherosclerotic lesion (arrows) on the inner wall.

In the hyperlipidemic rabbit, *in vivo *80 μm high-resolution T_1_-weighted CMR revealed a region of elevated signal intensity on the inner wall of the vessel extending into the lumen (Figure 8A, B), consistent with a pattern of fibrous atheroma tissue [29]. A small atherosclerotic lesion was confirmed in the corresponding histological section from the same location (Figure 8C). This shows a fairly homogeneous layer of fibrous tissue, with a higher lipid content rendering it paler than the adventitia. The area of the plaque region measured from CMR (Figure 8B) is 0.36 mm^2^, while the plaque area in the histological section is 0.33 mm^2^.

Visual inspection of the 5-10 mm sections harvested from the human vessel specimens and the rabbits prior to fixation revealed no evidence of vessel perforation, heat injury or plaque rupture attributable to the intravascular CMR detector.

## Discussion

In this paper we evaluated the utility of a high-resolution (80-μm) receive-only intravascular loopless CMR detector as a possible guidewire for identifying imaging and measuring the fibrous cap thickness of atherosclerotic plaques in a widely-available clinical 3T MR system. In a 1-cm region (*r ≤ *5 mm), potentially relevant to most cases of coronary and carotid vessel imaging, the detector retains over 17% of the peak sensitivity at its junction. Moreover its extended spatial sensitivity along a lead length of about double the whip length (Figures 1, 2, 6), permits angiographic imaging using relatively low-resolution coronal CMR to identify suspected atherosclerotic lesions, and supports a guidewire function under CMR alone that does not require X-ray fluoroscopy. This strategy differs from the CMR endoscopy approach [36,37] where the detector's intrinsic sensitivity is limited to a thin sensitive disk by design, and the CMR's frame-of-reference is locked to the probe-head to provide a "probe's-eye view". Here we have shown that, after detection by low-resolution angiographic-type CMR, suspect lesions can subsequently be targeted for high-resolution CMR that visualizes calcifications as signal voids (Figure 3) and permits accurate characterization of FCT (Figures 5, 6), a key predictor of plaque vulnerability to rupture.

Although the FCT measurements were only performed on human specimens in an electrically bio-analogous saline phantom, the feasibility of the approach was demonstrated with a 0.76 mm maximum diameter biocompatible nitinol loopless antenna guidewire *in vivo *in the rabbit aorta (Figures 7, 8). In these studies, artifacts from respiratory and cardiac motion were relatively minor, perhaps reflecting the limited spatial extent of high-resolution imaging. At least motion did not seem to affect the accuracy of measurements of aortic wall thickness or plaque size in T_2 _or T_1_-weighted CMR, which agreed with histological measurements to within 2% and 10%, respectively. The scan time for 3D cardiac-gated *in vivo *80 μm high-resolution CMR with the 3T loopless detector was 7-10 min (Figures 7, 8), which is within a range that is potentially tolerable for clinical research studies.

The improved performance of the 3T intra-vascular loopless detector compared to prior 1.5T devices derives from an approximately quadratic SNR performance with MR field-strength [33]. Here, the SNR gain was exchanged for a high-resolution intravascular imaging capability, and a higher-speed lower-resolution angiographic-type CMR that permitted device tracking. In addition, the extended CMR-sensitivity of the guidewire about the cable/whip junction obviated the need for precise device positioning or repeated tracking-scans, enabling high-resolution imaging anywhere within the detector's sensitive region. At 80 μm resolution, the 3T loopless detector provided exceptional views of the vessel wall and pathology (Figures. 3, 4, 7, 8), with FCT measurements accurate to within 13 ± 9% on average compared to histology, with which they were strongly correlated (Figure 5). This was also true of plaques with a mean FCT of less than the 650 μm threshold for discriminating symptomatic from asymptomatic carotid artery stenoses, as identified by IVUS [13]. The implication is that interventional 3T CMR with a loopless guidewire detector could potentially do the same.

Degrading the CMR spatial resolution negatively affected the accuracy of FCT measurements. Increasing the voxel size from 80 μm to 140 μm resulted in an over-estimate of 360 μm being recorded for a plaque with a 186 μm FCT measured by histology, possible distortions introduced during histological preparation notwithstanding. That an 180 μm cap is even observable with a 140 μm voxel size attests to the high contrast sensitivity of CMR, and again supports its potential for plaque detection at a coarser but more time-efficient spatial resolution, followed by high-resolution CMR for plaque characterization. Note also that the averaging of many measurements of FCT from high-resolution CMR can result in reasonably accurate estimates of FCT right down to the native resolution of the scans (Figure 5).

Nevertheless, the 80 μm resolution employed here is higher than the 65 μm FCT proposed for classifying fibrous caps in coronary plaques as vulnerable [7-10]. In the context of other intravascular imaging modalities, the CMR resolution achieved here is comparable to the best achieved by IVUS at around 100 μm [9,11-13]. However, as shown in prior studies comparing CMR with IVUS [14,29], CMR has better soft-tissue contrast and is not limited by the presence of calcifications [14,29], which were common in our sample cohort (eg, Figure 3). We could not match OCT's resolution of 10-20 μm [9,10], but CMR's FOV or penetration depth was a good order-of-magnitude larger than in OCT. Also, unlike either of these modalities, intravascular CMR did not require X-ray fluoroscopic guidance. Our scan-times for high-resolution CMR in the aorta were exacerbated by the use of cardiac-gated acquisition. Intravascular CMR of vessels further from the heart might benefit from more-efficient steady-state sequences if gating could be omitted. Also, the promise of a quadratically increasing SNR afforded by even higher B_0 _human MR scanners [33] offers further hope for 65 μm FCT measurements and/or much shorter scan-times.

## Conclusion

In conclusion, the purpose of the present work was to establish the feasibility of 3T intravascular CMR for quantifying FCT and to develop a strategy for its use *in vivo*. We found that a 3T loopless intravascular CMR detector could locate atherosclerotic lesions in human arterial specimens *in vitro*, accurately measure FCT at high-resolution over a range of about 100-1000 μm, and that the same approach is feasible *in vivo*. While it is hoped that 3 T intravascular CMR may provide a useful imaging option for quantitatively assessing vulnerable plaque and evaluating the efficacy of therapies designed to reduce plaque burden, further work will be required to determine its role in the context of existing intravascular imaging approaches.

## Abbreviations

CMR: cardiovascular magnetic resonance; FCT: fibrous cap thickness; SNR: signal to noise ratio; FOV: field of view; T_1_: spin-lattice relaxation time; T_2_: spin-spin relaxation time; H&E: hematoxylin and eosin (stain); OD: outer diameter; 2D: 3D: two: three dimensional; TSE: turbo spin-echo; FFE: fast field echo; TR: repetition time; TE: echo time; FA: flip-angle; IVUS: intravascular ultrasound; OCT: optical coherence tomography; RF: radio frequency.

## Competing interests

PAB has a financial relationship with MRI-Interventions, Inc (formerly Surgi-Vision, Inc) from whom the obsolete *Intercept *1.5T guidewire was obtained. The relationship is managed in accordance with Johns Hopkins Office of Policy Coordination.

## Authors' contributions

DQ and PAB both contributed to the concept, design, data interpretation, and the drafting and revision of the manuscript. DQ fabricated the experimental devices, conducted the experiments and collected the data. Both authors have read and approved the final manuscript.
